# Regulation of the tumor suppressor PML by sequential post-translational modifications

**DOI:** 10.3389/fonc.2012.00204

**Published:** 2012-12-31

**Authors:** M. Lienhard Schmitz, Inna Grishina

**Affiliations:** Department of Biochemistry, Medical Faculty, Justus Liebig University, German Center for Lung ResearchGiessen, Germany

**Keywords:** promyelocytic leukemia, PML-RARα, post-translational modifications, protein kinases, ubiquitin E3 ligases

## Abstract

Post-translational modifications (PTMs) regulate multiple biological functions of the promyelocytic leukemia (PML) protein and also the fission, disassembly, and rebuilding of PML nuclear bodies (PML-NBs) during the cell cycle. Pathway-specific PML modification patterns ensure proper signal output from PML-NBs that suit the specific functional requirements. Here we comprehensively review the signaling pathways and enzymes that modify PML and also the oncogenic PML-RARα fusion protein. Many PTMs occur in a hierarchical and timely organized fashion. Phosphorylation or acetylation constitutes typical starting points for many PML modifying events, while degradative ubiquitination is an irreversible end point of the modification cascade. As this hierarchical organization of PTMs frequently turns phosphorylation events as primordial events, kinases or phosphatases regulating PML phosphorylation may be interesting drug targets to manipulate the downstream modifications and thus the stability and function of PML or PML-RARα.

## Introduction

The PML protein has attracted the attention of many researchers as it is involved in a variety of diseases and it is able to form subnuclear structures. These are referred to as PML nuclear bodies (PML-NBs) but are also known as PML oncogenic domains, nuclear dot 10 or Kremer bodies (Bernardi and Pandolfi, 2007). Interphase cells typically contain between 10 and 20 PML-NBs in the nucleus, but their number and distribution varies considerably depending on cell type, cell cycle phase, and stress conditions. The human PML gene is located on chromosome 15q22 and alternative splicing of the C-terminal exons leads to the generation of 6 nuclear isoforms and a further isoform called PML-VII that is purely cytoplasmic (Jensen et al., 2001; Bernardi and Pandolfi, 2007). All these PML isoforms share the first three exons, which encode the RBCC/TRIM motif. This tripartite structure contains a RING (really interesting new gene) zinc-finger, two additional zinc-finger motifs (B-box1 and B-box2) and a DUF 3583 domain containing a coiled-coil region (Borden et al., 1995; Jensen et al., 2001). The TRIM motif -together with unique C-terminal domains of PML-II and PML-V allows self-assembly. PML proteins function as scaffold proteins by assembly to spherical PML-NBs containing the structure-forming PML and Sp100 proteins that are organized into patches within a 50–100-nm-thick shell (Lang et al., 2010; Geng et al., 2012). High-resolution 4Pi fluorescence laser-scanning microscopy studies complemented with correlative electron microscopy allowed a more detailed view on the substructure of PML-NBs which have an outer shell, while the inner part is accessible for diffusing nuclear factors (Lang et al., 2010). The inner part of PML-NBs builds a bioincubator containing densely packed proteins with exceedingly high concentrations. Molecular crowding within such PML-NB cages slows down diffusion, facilitates stochastic interactions, and makes them more efficient (Cho and Kim, 2012). High protein concentrations can thus increase the rates of intermolecular interactions by several orders of magnitude (Minton, 2001). In that sense PML-NBs function as bioincubators that allow or speed up PTMs of their permanent or transiently contained resident proteins. In addition to their scaffolding function, PML proteins may also harbor catalytic activities. PML functions as a SUMO (small ubiquitin-like modifier) E3 ligase for interaction partners such as p53 and Mdm2, as revealed by *in vivo* and *in vitro* experiments (Chu and Yang, 2011). The TRIM family member PML may also function as an ubiquitin E3 ligase, as suggested by the recently identified ubiquitin E3 ligase activity for the related TRIM16 protein. Although TRIM16 lacks a classical RING domain, autoubiquitination occurs through its B-boxes that can complex zinc atoms (Bell et al., 2012). It will now be relevant to see whether also PML has the ability to augment ubiquitination via its B-boxes. While most of the PML proteins reside in the nucleus, a fraction of PML is also found in the cytoplasm (Condemine et al., 2006; Jul-Larsen et al., 2010). In addition, the splicing variant PML-VII lacks the NLS (nuclear localization signal) and is thus exclusively found in the cytosol. The functional relevance of cytoplasmic PML was revealed in a seminal study that showed its contribution for transforming growth factor beta (TGFβ)-induced growth arrest and senescence. The necessity of cytoplasmic PML for TGFβ-mediated signaling also relies on the ability of PML to bind to the transcription factors Smad2/3 (Lin et al., 2004). A further study showed that PML can also localize to specific membrane structures that connect mitochondria with the endoplasmic reticulum. These membrane regions control apoptosis through the regulation of calcium influx from the ER to the mitochondria in a PML-dependent fashion. Accordingly, PML^−/−^ cells were protected from cell death induced by stimuli that rely on changes in Ca^2+^ signaling (Giorgi et al., 2010), showing that PML also plays an important role outside from the nucleus.

Various PTMs including phosphorylation, acetylation, ubiquitination, ISGylation, and SUMO modification can affect virtually all protein functions. Most PTMs are added or removed by specialized enzymatic machineries which results in complex PTM patterns that can be regulated in a signal- and cell type-specific fashion (Jensen, 2006). The activities of PTM-mediating enzymes such as protein kinases are themselves regulated by PTMs, thus creating highly wired signaling networks. More than 1000 different proteins serve as kinases or ubiquitin E3 ligases, providing an impressing illustration for the complexity of these networks (Manning et al., 2002; Deshaies and Joazeiro, 2009). The signaling proteins do not work as single independent units but rather function in dynamically interacting protein complexes. Most signaling proteins of the numerous pathways known to be regulated by PML occur in one or several stable or dynamic complexes. A number of examples show that a given protein can, depending on its molecular micro-environment and binding partners within a given multi-protein signaling complex, serve different functions (Vousden and Prives, 2009). The formation of multi-protein assemblies, their stability, and enzymatic activity are tightly controlled by PTMs, which act as reversible molecular switchboards. Although not every single PTM might be involved in a specific function (Lienhard, 2008), the strong overrepresentation of mutations in PTM modifiers (such as kinases or ubiquitin E3 ligases) in diseases such as cancer (Rikova et al., 2007; Puente et al., 2011) argues for their relevance for cellular homeostasis.

The correct assembly of PML-NBs facilitates protein phosphorylation and the modification of lysines through attachment of acetyl groups, ubiquitin chains, or ubiquitin-related peptides such as SUMO or ISG15 (interferon-stimulated gene-15). The analysis of a manually curated PML-NB interactome (Van Damme et al., 2010) allowed the identification of many proteins involved in SUMO modification, ubiquitination, phosphorylation, and acetylation. An updated list is displayed in Table 1. Given the large repertoire of PTMs it will be interesting to see whether PML-NBs are hotspots that are specialized for the above mentioned PTMs or whether also other types of protein modifications are enriched in these factories. As arginine methyltransferase 1 (PRMT1) is also found in PML-NBs (Boisvert et al., 2005) it will be relevant to investigate whether also arginine methylation is occurring in these subnuclear structures. The importance of PML for PTMs is nicely exemplified for the tumor suppressor p53. Expression of oncogenic Ras induces PML-mediated senescence in a p53-dependent manner. Ras also triggers recruitment of p53 and the acetyltransferase CREB-binding protein (CBP) to PML-NBs which results in the formation of a trimeric p53/PML/CBP complex and p53 Lys382 acetylation (Pearson et al., 2000). Ras-induced p53 acetylation and senescence are lost in PML^−/−^ fibroblasts, showing the relevance of PML for this critical process. Further insight for the involved mechanism was provided by the finding that p53 Lys382 acetylation requires previous phosphorylation of p53 at Ser46 by the kinase homeodomain-interacting protein kinase 2 (HIPK2) 2 (Hofmann et al., 2002). HIPK2-mediated Ser46 phosphorylation depends on the presence of the PML protein (Moller et al., 2003), explaining why also the associated p53 acetylation is PML-dependent. Acetylation of p53 is also impaired in cells expressing the oncogenic and dominant negative PML-RARα fusion protein which is causative for acute promyelocytic leukemia. This mechanism is dependent on histone deacetylases (HDACs) and the deacetylated p53 protein has a diminished activity, thus losing a substantial part of its tumor-suppressing function (Insinga et al., 2004). But also casein kinase 1 (CK1)-mediated phosphorylation of p53 at Thr18 is regulated by PML. DNA damage causes the partial recruitment of CK1 in PML-NBs where the kinase interacts with endogenous p53 and PML, thus enhancing p53 Thr18 phosphorylation (Alsheich-Bartok et al., 2008). The importance of PML for p53 function is not only restricted to the wildtype protein (Bao-Lei et al., 2006), but is also seen for gain-of-function p53 mutants (Haupt et al., 2009).

**Table 1:** **Post-translational modifiers in PML-NBs**.

**Kinases**	**Ubiquitin conjugating proteins**
Mtor	RBCK1
p38	FBX3
HIPK1	RNF63
HIPK2	TRIM69
CK2	TRIM27
CHEK2	SKP1
CK1	Cullin 1
Aurora kinase A	Cullin 3
MNK2b	E6AP
ATR	CHFR
DAPK3	RNF4
IKKε	TOPORS
AKT	KLHL20
**Phosphatases**	**Ubiquitin deconjugating proteins**
PP1A	USP45
PP2A	USP7
**Acetylation**	**SUMO conjugation**
CBP	HDAC7
p300	AOS1
Tip60	UBA2
	UBC9
**Deacetylation**	RANBP2
HDAC1	RNF4
HDAC2	TOPORS
HDAC3	MEL-18
HDAC7	p14ARF
Sirt1	PIAS1
	PIAS3
	PIASy
**Other enzymes**	PC2
Pin1	PIAS2/x
PRMT1	**SUMO deconjugation**
	SENP1-3
	SENP5-7

A comprehensive list of PTM-regulating proteins and enzymes contained in PML-NBs. Based on a published and manually curated network of the PML-NB interactome (Van Damme et al., 2010) and an updated literature review the PTM-regulating proteins are grouped according to the type of protein modification.

PML also modulates the activity of protein phosphatases, as seen for the protein phosphatases 1A and 2A (PP1A and PP2A). PML recruits the AKT phosphatase PP2A and the phosphorylated AKT (also known as PKB) protein into PML-NBs, thus ensuring that the majority of AKT is present in the unphosphorylated, inactive form (Trotman et al., 2006). The absence of PML leads to an exaggerated AKT phosphorylation and concomitantly to tumorigenesis in the prostate, a tissue that is sensitive to the levels of phosphorylated AKT. At the molecular level, increased AKT phosphorylation leads to impaired Foxo3a-mediated transcription of proapoptotic and cell cycle inhibitory genes (Fei et al., 2009).

But PML also serves as a regulator of acetylation events, as revealed in a study that uncovered the contribution of PML for enhanced fatty acid oxidation in metabolically hyperactive breast cancer cells (Carracedo et al., 2012). This study showed the contribution of PML for the regulation of the acetylation status of the PPARγ coactivator 1A (PGC1A). PML expression leads to reduced PGC1A acetylation and thus increases its activity (Carracedo et al., 2012). As a result, PPAR signaling is enhanced which in turn promotes fatty acid oxidation and helps to meet the increased requirement for energy and biomass in highly proliferating cancer cells. Elevated fatty acid oxidation by the cooperation between PML and PPAR transcription factors is also physiologically relevant for the maintenance of hematopoietic stem cells (Ito et al., 2012).

A further PTM that is affected by PML-NBs is ubiquitination. The accessory KLHL20 protein is contained in a multisubunit ubiquitin E3 ligase complex and binds to death-associated protein kinase (DAPK), thus resulting in its ubiquitination which in turn allows its proteasomal degradation. Stimulation of cells with interferon leads to the enrichment of KLHL20 in PML-NBs, thereby separating KLHL20 from DAPK which stops ubiquitination and degradation of this kinase (Lee et al., 2010). Conversely, PML-NBs can also regulate the activity of deubiquitinating enzymes such as USP7. PML is required to inhibit USP7, thus preventing the deubiquitination of the tumor suppressing phosphatase PTEN. Disruption of PML-NB integrity by PML-RARα leads to the ubiquitination of PTEN and to aberrant localization of this phosphatase, thus facilitating cancer progression (Song et al., 2008).

An open question relates to the specificity of the pathways and PTMs regulated by PML-NBs. A previously published review by Rosa Bernardi and Pier Paolo Pandolfi stated that “PML-NBs have been implicated in the regulation of virtually every biological function” (Bernardi and Pandolfi, 2007). It is certainly true that a large plethora of signaling pathways is regulated by PML-NBs, but there seems to be an overrepresentation of stress-related signaling events that range from (virus) infection to DNA damage or metabolic stress (Bischof et al., 2002; Everett and Chelbi-Alix, 2007; Tavalai and Stamminger, 2009; Carracedo et al., 2012). This would also be consistent with the phenotype of PML-deficient mice that are largely normal under convenient laboratory conditions, but show a variety of defects when challenged with adverse agents (Bernardi and Pandolfi, 2007; Borden, 2008). Given the recent advances in mass spectrometry protocols, instrumentation, and data analysis it will be exciting in the future to have a broader and more systematic view on the PTM patterns that are controlled by this subnuclear structure.

## Modifications of PML

Structural PML-NB proteins such as PML and Sp100 and also transiently contained residents undergo a variety of PTMs that will be discussed here. The necessity for regulated PTMs which in turn can regulate the functional properties of the modified proteins comes from some general considerations: (1) PML-NBs are dynamically regulated during the cell cycle by a fission mechanism during S phase (Dellaire et al., 2006a). PML-NBs disassembly during mitosis is paralleled by phosphorylation and de-SUMOylation of PML proteins and their dissociation from Sp100 (Everett et al., 1999; Dellaire et al., 2006b). In the G1 phase the PML-NBs show an increased association with sites of active transcription and enriched in hyperphosphorylated RNA Pol II (Kiesslich et al., 2002). (2) PML-NBs can be seen as integration and processing points for stress signals, as also discussed above. The differentiation between the presence or absence of these stress signals generally employs information processing steps that transmit and display signals through changes in PTM patterns. (3) It is tempting to speculate that the various PML-NBs contained in a given cell may be functionally heterogeneous and distinct, although experimental proof is missing. These (hypothetical) functionally distinct PML-NBs could exert overlapping but also specialized functions that could be defined by specific PTMs patterns. Collectively, these points argue for the necessity of basal and also signal-induced PTM patterns that allow the regulation of PML-NB (dis)assembly and function. Here we will discuss only modifications of PML and the PML-RARα fusion protein, but not of other important constituents of these subnuclear structures.

## PML phosphorylation

**Figure 1:**
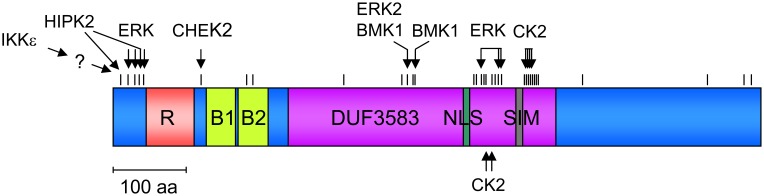
**Schematic display of the PML protein and its various domains.** The distribution and clustering of phosphorylation sites is shown, the directly phosphorylating kinases and their upstream regulators are indicated.

Phosphorylations very often have their own functions, but as discussed in detail below, this modification can be coupled to ubiquitination, SUMOylation, acetylation, and isomerization. Basal phosphorylation of PML was shown in one of the early and seminal papers in the PML field (Chang et al., 1995). Experimental work from many groups in combination with data retrieval from the public PhosphoSitePlus^©^ database allows the description of >30 PML phosphorylation sites that are schematically displayed in Figure 1 and also given in Table 2. Phosphorylation sites are strongly enriched in the N-terminal region preceding the RING domain. But they also cluster around the NLS and the SUMO-interacting motif (SIM), which potentially allows to control the subcellular localization of PML. The involved kinases and physiological consequences of PML phosphorylation are only known for the minority of phosphorylated sites. Modification of the N-terminus is mediated by several kinases including HIPK2. DNA damage induces HIPK2 activity and leads to direct PML phosphorylation at serines 8 and 38 as revealed by *in vitro* kinase assays (Gresko et al., 2009). These N-terminal phosphorylation sites further enhance DNA damage-induced PML SUMOylation and are required for the ability of PML to cooperate with HIPK2 for the induction of cell death. Ser38 phosphorylation is also observed after overexpression of IKKε, a kinase regulating innate immunity and DNA damage signaling (Renner et al., 2010). *In vitro* kinase assays showed that this IKKε-triggered phosphorylation is not direct, thus suggesting the involvement of a downstream kinase. Immunofluorescence experiments revealed that PML Ser38 phosphorylation is important for its ability to transiently recruit IKKε to PML-NBs (Renner et al., 2010). PML phosphorylation at Thr28, Ser36, Ser38, and Ser40 is mediated by extracellular signal-regulated kinase (ERK) after treatment with arsenic trioxide (As_2_O_3_). This phosphorylation is associated with increased SUMOylation of PML and elevated As_2_O_3_-induced cell death (Hayakawa and Privalsky, 2004). ERK2 also phosphorylates PML at Ser403 and Ser505 which allows docking of the prolyl-isomerase Pin1 which presumably causes isomerization of PML (Lim et al., 2011). Docking of Pin1 to PML then leads to destabilization of PML. Basal PML phosphorylation at Ser403 and Ser505 is detectable in unstimulated cells and can also be further induced by EGF stimulation (Lim et al., 2011), implying that this mechanism regulates PML levels under steady-state and inducible conditions. The phosphorylation site at Ser403 can be also mediated by BMK1/ERK5 (big MAP kinase). This kinase shows similarity to the ERK family and modifies PML at Ser403 and Thr409. Phosphorylation at these sites inhibits PML-dependent activation of p21 by an unknown mechanism (Yang et al., 2010).

**Table 2:** **List of PML phosphorylation sites retrieved from published literature and the public PhosphoSitePlus^©^ database**.

**Phosphorylation site**	**Kinase**	**PML domain**	**Biological effect**	**Conserved in mouse**
S8	HIPK2		+ apoptosis	Yes
T28	ERK		+ apoptosis	No
S36	ERK		+ apoptosis + PML-NB recruitment of IKKε	Yes
S38	HIPK2, ERK		+ apoptosis	Yes
S40	ERK		+ apoptosis	No
S117	CHEK2		+ apoptosis	No
T184, S190		B2 box		No/Yes
Y309, S399		DUF domain		Yes/Yes
S403	ERK2 BMK1/ERK5	DUF domain	+ Pin1-mediated PML degradation	Yes
S408		DUF domain		Yes
T409	BMK1/ERK5	DUF domain	− inhibition of PML-induced p21 induction	Yes
S480, T482, S504		DUF domain		Yes/No/Yes
S505	ERK2	DUF domain		Yes
S512	CK2	DUF domain		Yes
S518	CK2	DUF domain	+ tumor-suppression	Yes
S527	ERK	DUF domain		No
S530	ERK	DUF domain		Yes
S535		DUF domain		No
S560, S561, S562	CK2	Adjacent to SIM		Yes/Yes/Yes
S565	CK2		+ Pin1-mediated PML degradation	Yes
S578, S580, S583,				Yes/Yes/Yes
S625, S804, T867,				Yes/Yes/No
S879				No

The list also indicates the involved kinases, evolutionary conservation to mouse and the modified domain. Enhanced and diminished biological functions are indicated by + and −. The numbering of amino acids refers to full-length human PML-I.

Consistent with the function of PML-NBs in the DNA damage response, several PML phosphorylating kinases with a role in the DNA damage response are known. The kinase ATR (ataxia telangiectasia and Rad3 related) phosphorylates PML at an unknown site(s) which in turn results in the nucleolar translocation of PML and the sequestration of MDM2 (Bernardi et al., 2004). As MDM2 mediates constitutive ubiquitination of p53 and therefore limits its abundance, nucleolar sequestration of this E3 ligase leads to p53 stabilization and apoptosis. The kinase ataxia telangiectasia mutated (ATM) can also regulate the number of PML-NBs in response to DNA double-strand breaks, but this effect is probably independent from direct PML phosphorylation and rather depends on ATM-mediated phosphorylation of the KAP1 protein (Kepkay et al., 2011). DNA damage also activates checkpoint kinase 2 (CHEK2) which phosphorylates PML at Ser117. Functional experiments showed that induction of PML Ser117 phosphorylation is required for induction of cell death by γ irradiation (Yang et al., 2002). PML phosphorylation at Ser518 is mediated by casein kinase-2 (CK2), a kinase that is frequently found to be overexpressed in lung cancer. Consequently, a point mutated version of PML that is refractory to CK2 phosphorylation displays increased tumor-suppressive functions (Scaglioni et al., 2006). CK2-mediated PML phosphorylation also enhances PML ubiquitination and degradation, thus also allowing control of its abundance. *In vitro* kinase assays also revealed the ability of CK2 to phosphorylate a patch of serines directly adjacent to the C-terminal SIM (Stehmeier and Muller, 2009). These phosphorylations enhance the SUMO/SIM binding affinity and thus allow regulation of this interaction which is also important for the formation of PML-NBs (Lin et al., 2006; Shen et al., 2006; Sung et al., 2011).

## PML acetylation

Acetylation was detected in cells that were treated with the HDAC inhibitor trichostatin A (TsA). Similarly, PML acetylation was also induced by overexpression of the lysine acetyl transferases p300 or GCN5 (Hayakawa et al., 2008). Mapping experiments allowed the identification of Lys487 and Lys515 as the major acetylation sites in PML. A mass spectrometric analysis available from the public PhosphoSitePlus^©^ database confirmed acetylation at Lys487 and allowed the identification of Lys490 and Lys497 as additional modification sites (Hornbeck et al., 2012). PML acetylation is associated with increased PML SUMOylation, but the molecular mechanisms mediating this coupling effect are not understood. Cells expressing an acetylation-deficient mutant of PML were refractory to TsA-induced cell death (Hayakawa et al., 2008), but it is not clear whether this behavior can be attributed to an impairment of SUMOylation or acetylation. Interestingly, the acetylated Lys487 is contained in the NLS and thus acetylation of this residue can alter the localization of the modified PML protein and regulate its localization to the nucleus or cytoplasm. Deacetylation of this residue by SIRT1 is critical for nuclear localization of the circadian clock regulator Per2 (Miki et al., 2012). Accordingly, PML/PER2-mediated enhancement of BMAL1/CLOCK-mediated transcription is abolished upon mutation of Lys487, revealing the functional relevance of this acetylation site. As seminal studies showed that PML coimmunoprecipitates a significant level of HDAC activity, the majority of PML proteins will be in the non-acetylated state (Wu et al., 2001).

## PML SUMOylation

Modification of proteins with SUMO is carried out in a multi-step pathway. Similar to the conjugation of ubiquitin, the SUMOylation machinery also includes E1, E2, and E3 enzymes (Johnson et al., 1997). SUMO proteins are synthesized as precursors that need to be proteolytically processed by sentrin/SUMO-specific proteases (SENPs) to reveal a C-terminal diglycine motif. The mature SUMO is then activated in an ATP-dependent manner by the E1 enzyme (SAE2/Aos1) and catalyzes the formation of a thioester bond between the C-terminal glycine of SUMO and the catalytic cysteine residue of SAE2. SUMO is then transferred to the active site cysteine of the only known SUMO E2 conjugating enzyme Ubc9 (ubiquitin conjugating 9) (Johnson and Blobel, 1997). Finally, Ubc9 transfers SUMO to the target protein. This reaction results in an isopeptide bond between the C-terminal carboxy group of SUMO and the ε-amino group of the acceptor lysine. This process is typically facilitated by SUMO E3 ligases which are often SUMOylated themselves and/or contain a SIM (Yeh, 2009; Gareau and Lima, 2010). PML is among the first substrates that were found to be SUMO modified (Boddy et al., 1996; Sternsdorf et al., 1997). The early and pioneering work on PML SUMOylation has generated tools and knowledge that fueled the entire field of SUMO research. PML is SUMOylated at three lysines by the SUMO family members SUMO1, SUMO2, and SUMO3. PML can be SUMOylated at Lys65 in the RING finger, Lys160 in the B1-box, and Lys490 in the NLS (Kamitani et al., 1998). These modifications occur hierarchically as SUMOylation on Lys160 is a prerequisite for SUMOylation on Lys65. High-resolution microscopy enabled fascinating insights into the architecture of PML-NBs and allowed the detection of SUMO1 within aggregated spots in the PML-Sp100 protein shell (Lang et al., 2010). The SUMO1-containing patches also showed intrusions and extrusions from the PML shell. The differential colocalization between PML and SUMO1 may also reflect different degrees of PML SUMOylation. In contrast SUMO2 and SUMO3 occurred in the interior of PML-NBs (Lang et al., 2010). One important function of PML SUMOylation is the formation of normal nuclear bodies (Ishov et al., 1999; Zhong et al., 2000). Fluorescence recovery after photobleaching (FRAP) analysis showed that mutation of Lys160 and Lys490 drastically decreases the residence time of the mutant PML protein at the nuclear bodies (Weidtkamp-Peters et al., 2008). In support for the structural relevance of SUMOylation, PML-NBs are disrupted during mitosis as a result of de-SUMOylation (Everett et al., 1999). But PML-NB structure also relies on the ability of PML proteins to self-associate via the N-terminal RBCC domain (Borden, 2008). This self-association would also explain data showing that expression of a PML form mutated in all three SUMOylated lysines still allows the formation of PML-NBs (Lallemand-Breitenbach et al., 2001). The relevance of protein SUMOylation for PML-NB architecture is also supported by the analysis of SUMOylation-defective Ubc9^−/−^ cells which show PML-NB defects (Nacerddine et al., 2005). Stable assembly of PML-NBs also relies on the presence of a SIM domain which binds SUMO in a non-covalent manner. SIMs typically consists of a hydrophobic core, which is often flanked by acidic residues. SIM/SUMO interactions enhance or specify the interaction with other SUMOylated proteins. The relevance of the PML SIM for PML-NB formation has been challenged by the FRAP analysis of the PML isoform PML-VI which lacks a SIM. The exchange rate of PML-VI at PML-NBs was only marginally slower than exchange rates measured for SIM-containing PML isoforms (Weidtkamp-Peters et al., 2008). However, these studies were performed in cells containing endogenous PML, raising the possibility that the behavior of PML-VI could be influenced by association with endogenous PML forms harboring a SIM. The strength of SUMO/SIM interactions can be further regulated by CK2-mediated phosphorylation of a patch of serines directly adjacent to the C-terminal SIM (Stehmeier and Muller, 2009). These interactions can also be controlled by acetylation of SUMO which neutralizes basic charges and thus prevents binding to the SIM of PML. Expression of an acetylation-mimicking form of SUMO leads to a reduction in size and number of PML-NBs and also prevents recruitment of Daxx (death-domain associated protein) into these subnuclear structures (Ullmann et al., 2012). Pharmacological evidence suggests that class I HDACs antagonize SUMO acetylation, and it will be very interesting to identify the physiologically relevant signals regulating the acetylation status of SUMO in the future. Other functions of PML SUMOylation depend on the conjugated SUMO isoform and on the length of the poly-SUMO chain (Fu et al., 2005; Mukhopadhyay et al., 2006). While the importance for branched SUMO2/3 chains for subsequent ubiquitination of PML is discussed below, another report highlights the special relevance of oligomerized SUMO3 for nuclear localization of PML (Fu et al., 2005).

The SUMOylation status of PML can be regulated by various ways: these include enzymatic mechanisms executed by SUMO ligases and proteolytic SENP enzymes, non-enzymatic mechanisms such as the intracellular redox state (Bossis and Melchior, 2006) and probably also the relative availability and localization of unconjugated SUMO1 and SUMO2/3 (Lang et al., 2010). Basal SUMOylation is ensured by the direct binding of the PML RING finger to the SUMO E2 enzyme Ubc9 (Duprez et al., 1999). SUMO E3 ligases do not need a specialized catalytic center, as they function by bridging the SUMO-loaded Ubc9 and the substrate protein (Yeh, 2009; Gareau and Lima, 2010). This mechanism of induced proximity is sufficient to enhance SUMOylation and accordingly several enzymes have been proposed to function as SUMO E3 ligases for PML. Conjugation of SUMO2 (but not of SUMO1) is enhanced by RanBP2, suggesting that this E3 ligase functions as a paralog-specific enzyme (Tatham et al., 2005). Two further candidate enzymes have deacetylating functions: one is HDAC7, which shows increased PML binding in TNF-stimulated cells. The function as a SUMO E3 ligase was also recapitulated in a cell-free system with purified components, thus showing that this effect is direct (Gao et al., 2008). Another deacetylase is the NAD-dependent enzyme SIRT1 which stimulates PML SUMOylation in a deacetylase-independent manner (Campagna et al., 2011). Also the PIAS1 protein was identified as a PML-interacting protein with the ability to enhance PML SUMOylation, as revealed by *in vivo* and *in vitro* experiments (Rabellino et al., 2012). The relative specificity of these E3 enzymes is probably limited, as a given SUMO E3 ligase can typically enhance the SUMOylation status of several different substrate proteins. It will therefore be interesting to investigate SUMOylation of the endogenous PML protein in the absence of one or several of these discussed E3 ligases to reveal their relative contribution in different signaling pathways. Enzymatic de-SUMOylation of PML is mediated by several SENPs. SENP-1 decreases PML SUMOylation after treatment of cells with IL-6 (Ohbayashi et al., 2008). Transient transfection experiments showed that SENP-5 can remove SUMO2 or SUMO3 from Lys160 or Lys490, but it failed to remove SUMO1 from these two positions. On the other hand, SENP-5 expression resulted in the deconjugation of all three SUMO isoforms from Lys65 (Gong and Yeh, 2006). Poly-SUMO-modified PML is also a substrate of SENP6 which shows preference for SUMO polymers and is specific for SUMO2/3-modified substrates. Si-RNA-mediated depletion of SENP-6 results in an elevated number and size of PML-NBs and allows detection of increased PML SUMOylation (Tatham et al., 2005; Hattersley et al., 2011).

## PML ISGylation and ubiquitination

The PML-RARα fusion protein can be modified by the ubiquitin-related protein ISG15. Also conjugation of ISG15 occurs in an E1/E2/E3-dependent manner and ISGylation of the PML domain in PML-RARα has been observed after overexpression of the E1-like ubiquitin-activating enzyme for ISG15 (Shah et al., 2008). It will be interesting to identify the modified site and to measure the physiological regulation of this PTM. The conjugation of ubiquitin chains to proteins has different consequences. While attachment of Lys48-linked chains leads to subsequent proteasomal destruction of the modified protein, the attachment of non-Lys48-branched ubiquitin chains has regulatory consequences (Varshavsky, 2012). Similar to the SUMO system, also ubiquitination is an ATP-dependent process that proceeds by an E1, E2, and E3 cascade. Ubiquitin E3 ligases fall into several groups including the largest group of RING finger and RING finger-related E3s (Budhidarmo et al., 2012). Given the central relevance of PML as a highly connected hub that integrates and regulates a multitude of different signaling pathways it is not surprising to see that PML ubiquitination employs many different ubiquitin E3 ligases. Physiologically relevant ubiquitin-dependent degradation of PML occurs to control its half-life in unstimulated cells and also after stimuli such as hypoxia that lead to its degradation (Lallemand-Breitenbach et al., 2008; Tatham et al., 2008; Yuan et al., 2011). PML degradation and disruption of PML-NBs can be also exerted by the herpes simplex virus type 1 (HSV-1) immediate-early protein ICP0 (Cuchet-Lourenco et al., 2012). ICP0 directly interacts with PML-I and degradation of its interaction partner depends on an intact RING finger motif in this viral ubiquitin E3 ligase (Maul and Everett, 1994; Boutell et al., 2002; Gu and Roizman, 2003). Also hypoxia-mediated degradation of PML proceeds by a SUMO-independent pathway. Hypoxic conditions reduce the half-life of the PML protein and also lead to its increased ubiquitination (Yuan et al., 2011). Hypoxia leads to CDK1/2-mediated phosphorylation of PML at Ser518 by mechanisms that are poorly understood. This phosphorylation in turn creates a docking site for Pin1, which presumably causes conformational changes. The importance of Pin1 is underscored by the finding that Pin1 depletion also abrogates hypoxia-induced PML destruction. Prolyl-isomerization allows for subsequent recruitment of components of a multi-subunit ubiquitin E3 ligase complex (Yuan et al., 2011). The recruited components are Cullin-3 and the substrate adaptor KLHL20 which are required for hypoxia-induced ubiquitination of PML but also for steady state ubiquitination under normoxic conditions. The analysis of human prostate cancer showed that overexpression of KLHL20 and Pin1 correlates with down-regulation of PML, suggesting that this proteolytic pathway is also of pathophysiological relevance (Yuan et al., 2011). Another PML-destructing mammalian E3 ligase is E6AP (HPV E6-associated protein). Its overexpression causes the ubiquitination and proteasomal degradation of PML, while cells lacking this E3 ligase shows elevated PML levels. *In vitro* experiments with purified proteins showed that E6AP triggers the ubiquitination of PML (Louria-Hayon et al., 2009), which proofs that E6AP fulfills all criteria for a *bonafide* PML E3 ligase. The pathophysiological relevance of the E6AP-PML axis for development of B-cell lymphoma has been provided in mouse models where E6AP heterozygous mice were crossed with Eμ-myc transgenic mice which are a widely used model to study Burkitt lymphoma. E6AP heterozygosity resulted in a significantly delayed onset of Myc-induced lymphomagenesis. Lymphoid cells from Eμ-myc/E6AP^+/−^ mice showed increased levels of PML and concomitant elevated PML-mediated senescence (Wolyniec et al., 2012). The analysis of patient material showed increased E6AP expression in 60% of human Burkitt lymphomas. As down-regulation of E6AP in B-lymphoma cells restores expression of the tumor suppressor PML (Wolyniec et al., 2012), this ubiquitin E3 ligase would be an attractive drug target for the treatment of non-Hodgkin lymphomas. But this statement is also true for the other E3 ligases acting on PML, as it could restore expression of the tumor suppressor PML which is lost or reduced in many different human tumors (Bernardi and Pandolfi, 2007; Salomoni et al., 2012).

## Modifications of PML-RARα

### PML-RARα phosphorylation

While our knowledge on PML phosphorylation is steadily growing, less is known about this modification in the oncogenic PML-RARα fusion protein. Fusion of the *Pml* gene to the retinoic acid (RA) receptor α (*Rarα*) gene by the *t*(15,17) chromosomal translocation generates a fusion protein that preserves the N-terminal part of PML including its phosphorylation sites (De The et al., 1991; Kakizuka et al., 1991). Changes in the overall structure and localization of PML-RARα will presumably generate phosphorylation patterns that are distinct from the wildtype PML. But also differences in the kinetics can occur, as exemplified for DNA damage induced phosphorylation of Ser38 which is readily induced for PML and only occurs in a delayed fashion for PML-RARα (Gresko et al., 2009). RA is frequently used to treat PML and this agent also triggers growth arrest of leukemia-initiating cells (LICs). The cAMP-dependent phosphorylation of PML-RARα Ser873 is critical for PML-RARα degradation and subsequent clearance of LICs (Nasr et al., 2008). RA- or cAMP-initiated phosphorylation of the RARα moiety of the PML-RARα protein occurs at RARα Ser369. This phosphorylation can be exerted by MSK1 and allows cyclin-dependent kinase 7 (CDK7) to phosphorylate RARα at Ser77 (Gaillard et al., 2006; Bruck et al., 2009). Mutation of the Ser369 phosphorylation site in PML-RARα yields a protein with decreased sensitivity to RA-triggered degradation which results in resistance to RA (Nasr et al., 2008). These data show the relevance of PML-RARα phosphorylation for clinically relevant degradation of this oncogenic fusion protein, and emphasize the necessity to learn more about PML-RARα phosphorylation in the future.

### PML-RARα acetylation

Presently only indirect evidence suggests a functional role of PML-RARα acetylation for disease progression, as revealed by pharmacological evidence in a transgenic mouse model. PML-RARα expressing mice develop leukemia which is responsive to RA treatment. This mouse model recapitulates the function of PML-RARα as a transcriptional repressor and the repressor activity is lost upon treatment of cells with RA in combination with TsA (He et al., 1998). It will be interesting to study whether the TsA activity is attributable to increase acetylation of the PML-RARα protein or other factors. Acetylation occurs for lysines, residues that can be also modified by ubiquitination and SUMOylation. It is therefore possible that PML acetylation has also an impact on the other two modifications which are frequently found in competition for unmodified side chains of lysines (Li et al., 2012).

### PML-RARα SUMOylation

The PML-RARα fusion protein still contains two of the three PML SUMOylation sites at Lys65 and Lys160. Data obtained in cell models and transgenic animals show the necessity of the Lys160 SUMOylation site in the PML moiety of the fusion protein for the development of acute PML (Zhu et al., 2005). Lys160 SUMOylation is important for binding to the transcriptional repressor Daxx, raising the possibility that Daxx-mediated gene repression is important for leukemic transformation. SUMOylation of PML-RARα is also required for arsenic trioxide-induced degradation of PML-RARα. Several mechanisms lead to arsenic-triggered SUMOylation of PML-RARα, which is the prerequisite for its ubiquitin-mediated degradation as discussed below. Arsenic causes oxidative stress (Kawata et al., 2007), which facilitates the formation of disulfide bonds between PML proteins, thus resulting in PML multimerization and targeting to nuclear bodies (Jeanne et al., 2010). In addition, arsenic directly binds to PML cysteines, which allows enhanced binding of Ubc9 and elevated PML SUMOylation (Jeanne et al., 2010; Zhang et al., 2010). Arsenic causes the modification of PML/ RARα at Lys65 and Lys160 by mixed poly-SUMOylation chains containing all three SUMO isoforms (Lallemand-Breitenbach et al., 2008; Tatham et al., 2008; Weisshaar et al., 2008). The SUMOylation state of PML-RARα is also regulated by E3 ligases such as PIAS1 (Rabellino et al., 2012), but is not clear whether E3 ligases or accessory proteins contribute to the arsenic-induced SUMOylation.

### PML-RARα ubiquitination

Of special pathophysiological interest is the ubiquitin-dependent degradation of PML-RARα by two clinically active drugs, arsenic, and RA. A SUMO-ubiquitin-cascade has been identified by several groups as the molecular mechanism leading to the arsenic-mediated degradation of PML-RARα (Lallemand-Breitenbach et al., 2008; Tatham et al., 2008; Weisshaar et al., 2008). The initial arsenic-induced Lys160 poly-SUMOylation creates docking sites for the ubiquitin ligase RNF4 (RING finger protein 4) which harbors 4 SIMs and thus can be anchored on these mixed chains. RNF4 in turn can decorate PML by Lys48-branched ubiquitin at Lys401 and probably further sites and thus cause its proteasomal elimination. RNF4 cannot discriminate between SUMO paralogs, raising the possibility that also mixed poly-SUMO chains function in the PML degradation pathway (Tatham et al., 2008). This mechanism does not only occur for PML-RARα but is principally similar in PML which is also degraded by arsenic. As PML SUMOylation is the primary event that is followed by polyubiquitination, the initial SUMO modification is the decisive and critical step. Accordingly, mutation of the SUMOylation site at Lys160 renders the mutated PML protein resistant to arsenic-induced degradation (Lallemand-Breitenbach et al., 2008). While these elegant studies revealed a detailed mechanism for arsenic-triggered PML-RARα degradation, the mechanisms mediating RA-induced PML-RARα elimination are less clear. This proteolytic pathway is dependent on transcriptional activation and requires DNA binding and the ligand-dependent transactivating domain (Zhu et al., 1999). This RA-induced transcriptional program results in the proteasome-mediated destruction of PML-RARα, but the involved molecules and ubiquitination events remain unclear. Two further family members of RING ubiquitin E3 ligases have been identified as mediators of PML-RARα degradation: SIAH1 and SIAH2 (seven in absentia homolog 1/2). Overexpression of either SIAH1 or SIAH2 leads to the ubiquitin/proteasome-dependent degradation of PML-RARα (Pietschmann et al., 2012), but it is currently not known whether this process controls basal or signal-induced degradation of PML-RARα.

## Outlook

**Figure 2:**
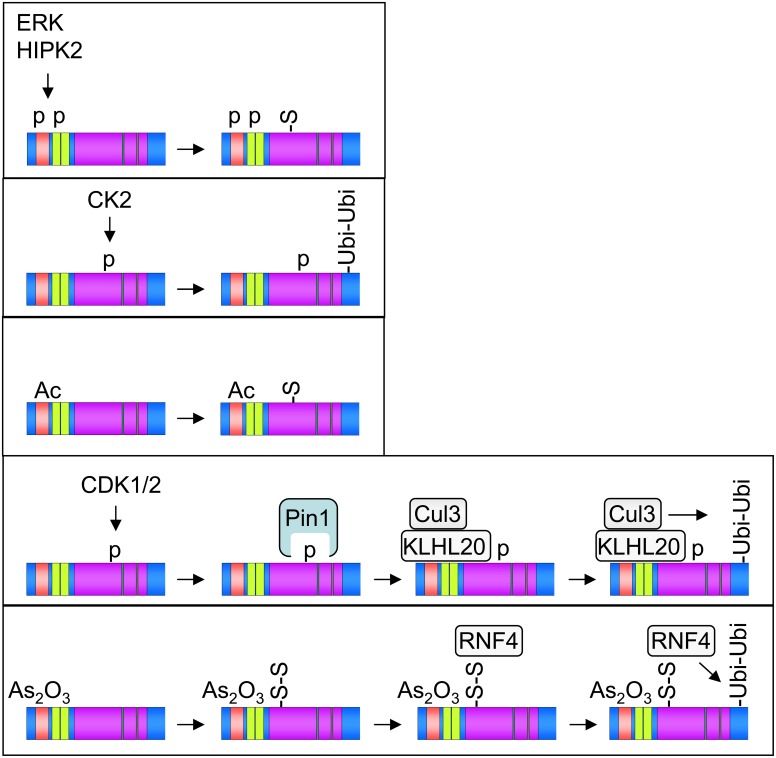
**Selected examples for the occurrence of hierarchical and combinatorial PML PTMs.** Phosphorylation (p), acetylation (Ac), mono- and poly-SUMOylation (S and S-S), and polyubiquitination (Ubi-Ubi) are shown, the involved enzymes are displayed.

Apparently, many of the PML modifications occur in hierarchical and timely organized fashion, as schematically summarized in Figure 2. Phosphorylation or acetylation events constitute typical starting points of many PTM cascades, while degradative ubiquitination is a characteristic irreversible end point. How can it be that PML is targeted by so many different modifying enzymes? Given its involvement in a plethora of biological functions it is certainly one of the central signaling hubs that needs to be controlled and fine-tuned by many different signaling pathways. The rapid development of mass spectrometric methodology and instrumentation will allow the identification of more and probably also novel PTMs in PML-NBs. It will also be relevant to clearly distinguish between PTM patterns that occur under unstimulated basal steady-state conditions or in response to specific signals in a more systematic fashion. Mass spectrometry can also clarify whether some PML modifications are restricted to specific cells or tissues which would open further avenues for research. Future work will also identify more of the transiently recruited PML-NB modifiers that serve to regulate the modification status of PML. It will be interesting in future experiments to quantify the occurrence and regulation of PML modification using stable isotope labeling by amino acids in cell culture (SILAC) experiments. Some modifications such as phosphorylation or acetylation can be detected with modification-specific antibodies, thus enabling the determination of individual modifications for endogenous PML proteins. These antibodies would also be instrumental to analyze the possible occurrence of specialized subgroups of PML-NBs. The functional role of individual modifications can be revealed by the analysis of knockin mice that express PML variants that are mutated in individual modification sites.

### Conflict of interest statement

The authors declare that the research was conducted in the absence of any commercial or financial relationships that could be construed as a potential conflict of interest.
